# Surgical experience and patient-related restrictions predict the adequacy of cervical mediastinoscopy in non-small cell lung carcinoma lymph node staging

**DOI:** 10.1186/s13019-018-0821-7

**Published:** 2018-12-29

**Authors:** Theo J. Klinkenberg, Wobbe Bouma, Caroline Van De Wauwer, Rienhart F. E. Wolf, Massimo A. Mariani, Harry J. M. Groen

**Affiliations:** 1 Department of Cardiothoracic Surgery, University of Groningen, University Medical Center Groningen, Groningen, The Netherlands; 2 Department of Pulmonary Diseases, University of Groningen, University Medical Center Groningen, Groningen, The Netherlands

**Keywords:** Mediastinoscopy, Video-assisted mediastinoscopy, Non-small cell lung cancer, Experience

## Abstract

**Background:**

Until recently, cervical mediastinoscopy was considered to be the reference standard for mediastinal staging for Non-Small Cell Lung Carcinoma (NSCLC). In the absence of metastases, mediastinal lymph node involvement is the most important prognostic factor and as such it determines therapeutic strategies. In this study we evaluated the adequacy of cervical mediastinoscopy in NSCLC lymph node staging in a large university hospital over more than a decade. In addition, we determined the influence of: (1) surgeon’s experience (2) video-assisted mediastinoscopy (VAM) and (3) patient-related restrictions (PRR) on the adequacy of lymph node sampling.

**Methods:**

Between January 2001 and December 2014, 225 patients underwent cervical mediastinoscopy for lymph node staging. Surgical and histological data were reviewed. Thirty-day follow-up was available for all patients. Lymph node sampling was considered adequate when stations 4 L, 4R and 7 were sampled (ESTS guidelines). A surgeon was considered to be experienced when he or she performed at least 40 procedures during the study-period.

**Results:**

Intraoperative mortality was 0%. Thirty-day mortality was 1.3%. Overall adequacy of lymph node sampling was 56%. Univariate and multivariate logistic regression analyses of lymph node sampling adequacy revealed level of surgical experience and PRR as independent predictors of lymph node sampling adequacy.

**Conclusions:**

Surgical experience and PRR independently predict the adequacy of cervical mediastinoscopy in NSCLC lymph node staging. VAM does not independently predict the adequacy of mediastinal lymph node sampling. In light of the expected further decline in mediastinoscopy numbers, we recommend to limit this procedure exclusively to the armamentarium of the experienced thoracic surgeon.

## Background

Lung cancer is the leading cause of cancer death in developed countries and accounts for an estimated 20% of all cancer deaths [1]. Five-year survival can be achieved in 40–50% of patients with early stage non-small cell lung carcinoma (NSCLC) [2]. Accurate staging based on tumour size, regional lymph node involvement and presence of metastasis is essential for treatment of NSCLC patients [3–5]. In the absence of metastases, mediastinal lymph node involvement is the most important prognostic factor and determines therapeutic strategies; i.e. patients with mediastinal nodal disease will in general not benefit from upfront surgery [6, 7].

FluoroDeoxyGlucose - Positron Emission Tomography – Computed Tomography (FDG-PET-CT), Endoscopic UltraSound guided-Fine Needle Aspiration and EndoBronchial UltraSound guided-TransBronchial Needle Aspiration (EUS-FNA/EBUS-TBNA) have become the most important techniques in mediastinal lymph node assessment in recent years [8, 9]. Cervical mediastinoscopy was considered to be the reference standard for mediastinal staging of lung cancer. After its introduction in 1957 mediastinoscopy has evolved considerably [10]. Video-assisted mediastinoscopy (VAM) was first reported in literature in 2002 [11] and was introduced at our center in September 2008 and from then on used in each mediastinoscopy case. VAM improved visualization and facilitated teaching tremendously [12]. However, no difference in sensitivity or negative predictive value was found when compared to conventional mediastinoscopy [13]. Nevertheless, the revised ESTS guidelines recommend VAM over conventional mediastinoscopy because of its superior visualization and safety [8].

Mediastinoscopy provides access to the upper paratracheal lymph nodes (stations 2R and 2 L), the lower paratracheal lymph nodes (stations 4R and 4 L) and subcarinal lymph nodes (station 7) [8, 14, 15]. The European Society of Thoracic Surgeons (ESTS) guidelines recommend to acquire at least samples from the lower paratracheal lymph nodes (stations 4R and 4 L) and the subcarinal lymph nodes (station 7) [8]. If present, the upper paratracheal lymph nodes should also be biopsied [8].

A well-executed cervical mediastinoscopy has a sensitivity of 76–85% and a negative predictive value of 82–92% [16] with an overall morbidity of 1.07% and mortality of 0.05% [17]. It is however important to realize that these values are largely dependent on the level of experience of the surgeon and the extensiveness of lymph node sampling [18]. Therefore, in daily practice, the actual adequacy and reliability of cervical mediastinoscopy is expected to be lower.

In this study we evaluated the adequacy of mediastinal lymph node sampling at our center over more than a decade. In addition, we analyzed the influence of: (1) surgeon’s experience, (2) the use of VAM and (3) patient-related restrictions (PRR) on the adequacy of lymph node sampling (based on the ESTS guidelines).

## Methods

This study was conducted in accordance with the guidelines of the University Medical Center Groningen Institutional Review Board.

### Patients

Between January 2001 and December 2014, 225 patients underwent cervical mediastinoscopy for NSCLC lymph node staging. VAM was introduced at our center in September 2008 and from then on used in each mediastinoscopy case. Patient characteristics are summarized in Table 1. Surgical and histological reports were reviewed. Thirty-day follow-up of survivors was complete and no patient was lost to follow-up.

**Table 1:** Preoperative Patient Data (*n* = 225)

Variable^a^	Value
Age, years	62.4 ± 10.1
Sex	
Male	167 (74)
Female	58 (26)
Histology primary lung tumor	
Squamous cell carcinoma	118 (52)
Adenocarcinoma	59 (26)
Large cell carcinoma	45 (20)
Adenosquamous carcinoma	2 (1)
NSCLC not otherwise specified	1 (0)
Clinical N-status	
N0–1	116 (52)
N2	100 (44)
N3	9 (4)
Clinical Stage^b^	
IA	5 (2)
IB	7 (3)
IIA	12 (5)
IIB	45 (20)
IIIA	109 (48)
IIIB	35 (16)
IIIC	3 (1)
IVA	9 (4)
IVB	0 (0)
Purpose of cervical mediastinoscopy	
Staging	138 (61)
Staging of tumor with unknown histology	66 (29)
Restaging after chemotherapy	20 (9)
Restaging after earlier mediastinoscopy	1 (0)
Video cervical mediastinoscopy	187 (83)
Level of surgeon’s experience	
Experienced surgeon	129 (57)
Less experienced surgeon	96 (43)

^a^Data are presented as mean ± standard deviation or number (%)

^b^Based on the 8th edition of the TNM classification for lung cancer (International Association for the Study of Lung Cancer)

*NSCLC* non-small cell lung carcinoma

### Adequacy of lymph node sampling

Based on the ESTS guidelines the minimal requirement for adequate lymph node sampling during cervical mediastinoscopy was defined as histologically proven samples from at least the left and right lower paratracheal lymph nodes (station 4 L and 4R) and the subcarinal lymph nodes (station 7) [8].

### Level of surgical experience

The level of surgical experience was based on the number of cervical mediastinoscopies performed by individual surgeons. For surgeons who performed at least 40 mediastinoscopies during the study-period the adequacy of lymph node sampling was > 70%. Therefore experienced surgeons were defined as those who performed at least 40 mediastinoscopies during the study-period. Based on these criteria two out of sixteen surgeons could be considered experienced. Both experienced surgeons in this study were trained as thoracic surgeons.

### Patient-related restrictions (PRR)

PRR were defined as intraoperative conditions or findings, which complicated the adequacy of lymph node sampling. An overview of PRR is shown in Table 2.

**Table 2:** Intraoperative and Postoperative Patient Data (*n* = 225)

Variable^a^	Value
Mean number of sampled lymph node stations (per patient)	3.5 ± 1.2
Mean number of samples taken (per patient)	11.0 ± 7.3
Adequate lymph node sampling	
Based on the ESTS guidelines	127 (56)
Patient-related restrictions	20 (8.9)
Adhesions	7 (3.1)
Bleeding, imparing sight	4 (1.8)
Tumor growth into the mediastinum (inablity to reach all stations)	2 (0.9)
Adequate biopsy of very suspicious node (no further biopsies taken)	2 (0.9)
Patient habitus	1 (0.4)
Extremely limited neck mobility	1 (0.4)
No samples taken on left side due to pre-op hoarseness	1 (0.4)
Struma	1 (0.4)
Anomaly of the innominate vein	1 (0.4)
Intraoperative mortality	0 (0)
Thirty-day mortality	3 (1.3)
Post-operative complications	7 (3.1)
Permanent recurrent laryngeal nerve lesion	3 (1.3)
Bleeding (causing respiratory insufficiency and intubation)	1 (0.4)
Pneumonia	1 (0.4)
Pneumothorax treated with chest tube	1 (0.4)
Atrial fibrillation	1 (0.4)

^a^Data are presented as mean ± standard deviation or number (%)

*ESTS* European Society of Thoracic Surgeons

### Follow-up

Follow-up was obtained directly from outpatient visits or by telephone interview with the patient and/or the referring physician. Thirty-day follow-up was complete.

### Statistics

Continuous variables were expressed as mean ± SD. Categorical variables were expressed as percentages. Comparisons between groups were performed using Pearson’s X^2^ test or Fisher’s exact test as appropriate for categorical variables and the independent samples t-test or Mann-Whitney U test, as appropriate for continuous variables. Univariate variables with *P* < 0.10 were included in the multivariate analysis. Age and gender were forced in the multivariate model. Multivariate logistic regression analyses by means of a forward stepwise algorithm were performed to identify independent predictors of lymph node sampling adequacy. Odds ratios were reported with 95% confidence intervals (CI). Goodness-of-fit of the final logistic regression models was assessed with the Hosmer-Lemeshow statistic.

All calculations were performed using a commercially available statistical package (IBM SPSS Statistics 22.0; IBM Corporation, Armonk, NY). Statistically significant differences were defined as *P* < 0.05.

## Results

### Lymph node sampling adequacy and its predictors based on the ESTS guidelines

The overall adequacy of lymph node sampling was 56%. In patients who underwent cervical mediastinoscopy by an experienced surgeon, adequacy of lymph node sampling was 64%, versus 47% when operated by a less experienced surgeon *(P =* 0.013, Table 3). When PRR occurred, adequacy of lymph node sampling was 20%, versus 60% when these restrictions did not occur *(P =* 0.002, Table 3). The distribution of PRR was not different between patients operated by experienced or less experienced surgeons. PRR did not differ significantly between less experienced and experienced surgeons (PRR 7.8% vs. 10.4%, respectively and *P* = 0.489). Univariate and multivariate logistic regression analyses of lymph node sampling adequacy are shown in Table 3. Multivariate analysis revealed level of surgeon’s experience and PRR as independent predictors of lymph node sampling adequacy. The Hosmer-Lemeshow goodness-of-fit test was non-significant, indicating that this multivariate model is a good fit (X^2^ = 0.24, df = 1, *P* = 0.878).

**Table 3:** Predictors of lymph node sampling adequacy by univariate analysis and multivariate logistic regression

Variable	Univariate analysis			Multivariate analysis		
	OR	95% CI	*P* value	OR	95% CI	*P* value
Age, years	1.02	0.99–1.05	0.141	–	–	–
Female sex	1.51	0.81–2.79	0.192	–	–	–
Squamous cell carcinoma histology	1.14	0.96–1.36	0.147	–	–	–
Video cervical mediastinoscopy	0.40	0.19–0.87	0.021	–	–	–
Experienced surgeon	1.98	1.16–3.39	0.013	1.96	1.13–3.41	0.017
No patient related restrictions	6.00	1.94–18.59	0.002	5.94	1.90–18.60	0.002

### Thirty-day mortality and post-operative complications

An overview of thirty-day mortality and post-operative complications is provided in Table 2. Thirty-day mortality was 1.3% (*n* = 3). All deaths were unrelated to cervical mediastinoscopy. Causes of death included cerebrovascular accident and respiratory insufficiency after partial mandibular resection for a second primary tumour, respiratory insufficiency after thoracotomy and rib resection, and multi-organ failure after early bronchial fistula formation following right-sided pneumonectomy.

## Discussion

This study demonstrates that surgical experience as well as PRR are independent and powerful predictors of the adequacy of cervical mediastinoscopy in NSCLC lymph node staging. When an experienced surgeons performs the mediastinoscopy adequate lymph node sampling is almost 2 times more likely than when a less experienced surgeon performs the mediastinoscopy (OR 1.96) and when PRR are not present adequate lymph node sampling is almost 6 times more likely than when PRR are present (OR 5.94). Other studies have also shown that mediastinoscopy yield depends strongly on operator skills [18, 19] and lymph node location [20]. The most frequent PRR in this study included adhesions, bleeding (impairing sight), and tumor growth into the mediastinum (inability to reach all lymph node stations). Although PRR did not differ significantly between less experienced and experienced surgeons, one might assume that a more experienced surgeon might be able to overcome certain PRR more easily than a less experienced surgeon. However, our data do not support this assumption. Both surgical experience and PRR proved to be independent predictors in multivariate analysis.

One of the drawbacks of conventional mediastinoscopy is the uncomfortable position for the surgeon. The surgeon has only a narrow view through the instrument and has to find a way among anatomical entities such as; trachea, esophagus, azygos vein, right pulmonary artery, recurrent nerve and pleural space/lung, and depending on patient anatomy; the carotid and innominate arteries. As such, conventional mediastinoscopy is a complex procedure and teaching can also be difficult because of the risk of ‘collateral damage’. These events strongly depend on the experience and teaching skills of the surgeon. VAM, with its superior visualization and teaching possibilities, has made the procedure safer and easier to adopt for surgeons in training [21]. In this study the use of VAM was not an independent predictor of adequacy of lymph node sampling, which supports the general observation that the superior visualization with VAM does not lead to a higher quality of mediastinal lymph node sampling compared to conventional mediastinoscopy [13].

Successful treatment of patients with NSCLC strongly depends on strict and reliable staging. The mediastinal lymph node status determines the sequence of treatment modalities. Until recently, mediastinoscopy was the gold standard for invasive mediastinal lymph node staging in NSCLC. Mediastinoscopy provides access to upper paratracheal lymph nodes (stations 2R and 2 L), lower paratracheal lymph nodes (stations 4R and 4 L) and subcarinal lymph nodes (station 7) [14], and has limitations in assessing the posterior subcarinal, lower mediastinal, and hilar lymph nodes [22]. EBUS-TBNA and EUS-FNA have shown to be at least equivalent to mediastinoscopy in sensitivity and negative predictive value [16]. For that reason, and because of the minimally invasive character of these procedures, they are currently recommended to be first choice for invasive mediastinal lymph node staging in lung cancer [8]. EBUS-TBNA and EUS-FNA are safe procedures with minor complications, reported in less than 1% of cases [23, 24]. Especially the combination of EBUS-TBNA and EUS-FNA allows complete access to nearly all lymph nodes of the mediastinum [25, 26]. However, pathological assessment of the yield of both procedures is only possible by cytology instead of histology. The samples obtained by needle aspiration are non-diagnostic in a significant number of cases [27] and depend strongly on operator skills [22]. These non-diagnostic cases led to the development of Rapid On-Site Evaluation of the aspirate in order to increase accuracy. This is achieved by monitoring on-site microscopy of repeated lymph node aspirations in different directions of the node until representative samples have been obtained [28].

Limitations of our study include the long time frame and the retrospective design.

Both mediastinoscopy and endosonography are complex technical procedures and depend strongly on operator skills and experience. The complexity of a procedure is inversely related to the adoptability of a procedure [29]. Complexity and adoptability determine the steepness of the learning curve of a procedure and depends on the quantity of procedures performed by the operator. With the growing experience in endosonography, the quantity of mediastinoscopies performed for mediastinal staging in NSCLC is likely to fall back and with it, the adoptability. In this study, we have shown that surgical experience and PRR are key in adequate lymph node sampling. Therefore, in light of the expected further decline in mediastinoscopy numbers, we recommend to limit this procedure exclusively to the armamentarium of the experienced thoracic surgeon.

## Conclusions

Surgical experience and PRR are powerful and independent predictors of the adequacy of cervical mediastinoscopy in NSCLC lymph node staging. Experience and skills vary with the training of the operator. Therefore, a solid training is required in educational programs and every center has to look at its own diagnostic yield and negative predictive value. VAM with its superior visualization and teaching possibilities, makes the procedure safer and easier to adopt for surgeons in training, but does not independently predict the adequacy of lymph node sampling. Since mediastinal lymph node staging is crucial in patient treatment and outcome, we urge that cervical mediastinoscopy should be performed and taught by experienced thoracic surgeons only.

## Abbreviations

Ca: Carcinoma
CI: Confidence interval
EBUS-TBNA: EndoBronchial UltraSound guided-TransBronchial Needle Aspiration
ESTS: European Society of Thoracic Surgeons
EUS-FNA: Endoscopic UltraSound guided-Fine Needle Aspiration
FDG-PET-CT: FluoroDeoxyGlucose - Positron Emission Tomography – Computed Tomography
NSCLC: Non-small cell lung carcinoma
PRR: Patient-related restrictions
SD: Standard deviation
VAM: Video-assisted mediastinocopy
